# Ca^2+^-regulated cyclic electron flow supplies ATP for nitrogen starvation-induced lipid biosynthesis in green alga

**DOI:** 10.1038/srep15117

**Published:** 2015-10-09

**Authors:** Hui Chen, Jinlu Hu, Yaqin Qiao, Weixian Chen, Junfeng Rong, Yunming Zhang, Chenliu He, Qiang Wang

**Affiliations:** 1 Key Laboratory of Algal Biology, Institute of Hydrobiology, Chinese Academy of Sciences, Wuhan, China; 2 University of Chinese Academy of Sciences, Beijing, China; 3 SINOPEC Research Institute of Petroleum Processing, Beijing, China

## Abstract

We previously showed that both the linear photosynthetic electron transportation rate and the respiration rate dropped significantly during N starvation-induced neutral lipid accumulation in an oil-producing microalga, *Chlorella sorokiniana*, and proposed a possible role for cyclic electron flow (CEF) in ATP supply. In this study, we further exploited this hypothesis in both *Chlorella sorokiniana* C3 and the model green alga *Chlamydomonas*. We found that both the rate of CEF around photosystem I and the activity of thylakoid membrane-located ATP synthetase increased significantly during N starvation to drive ATP production. Furthermore, we demonstrated that the *Chlamydomonas* mutant *pgrl1,* which is deficient in PGRL1-mediated CEF, accumulated less neutral lipids and had reduced rates of CEF under N starvation. Further analysis revealed that Ca^2+^ signaling regulates N starvation-induced neutral lipid biosynthesis in *Chlamydomonas* by increasing calmodulin activity and boosting the expression of the calcium sensor protein that regulates Pgrl1-mediated CEF. Thus, Ca^2+^-regulated CEF supplies ATP for N starvation-induced lipid biosynthesis in green alga. The increased CEF may re-equilibrate the ATP/NADPH balance and recycle excess light energy in photosystems to prevent photooxidative damage, suggesting Ca^2+^-regulated CEF also played a key role in protecting and sustaining photosystems.

Biodiesel, one of the most commonly used biofuels, is an ideal recyclable energy carrier, and thus also a potential primary energy source^1^,^2^. Various biolipids can be used as feedstock for biodiesel production, including edible vegetable oils, non-edible oils, and microalgae oils^2^,^3^,^4^,^5^. There has recently been a renewed interest in producing biodiesel from microalgae^6^,^7^,^8^. Consequently, microalgae that can grow rapidly and convert solar energy into chemical energy via CO_2_ fixation are now considered a promising oil source for biodiesel production^9^.

Lipid accumulation occurs within microalgal cells and varies with growth conditions. For instance, nitrogen (N) limitation or starvation increases lipid accumulation^10^,^11^. When N supplies are insufficient to support the protein synthesis required for growth, excess carbon from photosynthesis is channeled into storage molecules, such as triglyceride or starch^12^. Various microalgal species were reported to accumulate between 40 and 70% of lipid per dry cell weight (DCW) under N limitation or starvation conditions^9^,^10^,^12^.

Enzymes and structural proteins depend on adenosine triphosphate (ATP), an end product of photophosphorylation and cellular respiration, to fuel biosynthetic reactions, motility, and cell division^13^. In green algae, two different pathways of electron transport during photosynthesis exist: linear electron flow (LEF) and cyclic electron flow (CEF)^14^. During oxygenic photosynthesis, photosystem (PS) I and II cooperate to achieve a LEF that produces reducing power (NADPH) and generates a transmembrane proton gradient that drives ATP biosynthesis. CEF around PS I only produces ATP^13^,^15^. The NADPH and ATP can be used in the Krebs and glyoxylate cycles to yield NADH for respiratory oxidative phosphorylation (ATP production) in the mitochondrion^13^. Most of the ATP needed by algae is provided by photophosphorylation and respiratory oxidative phosphorylation. Although lipid accumulation is an energy storage process, additional energy in the form of ATP is required to drive this biosynthetic process. However, the regulatory mechanism underlying lipid accumulation in oil-producing microalgae is complex, and the source of ATP that drives lipid accumulation under N starvation remains to be identified. We previously found that both the photophosphorylation and respiratory oxidative phosphorylation rates decrease during the N starvation induced oil droplet formation in *Chlorella sorokiniana* C3, while the rate of CEF increased, possibly producing the ATP needed for triacylglycerol (TAG) synthesis^16^. However, the mechanism underlying these metabolic changes was unclear.

As a ubiquitous intracellular secondary messenger, Ca^2+^ plays an important role in the signal transduction events occurring in response to environmental stimuli, such as light, temperature, CO_2_, O_2_, water, nutrients, and stresses in plants^17^. Furthermore, Ca^2+^ sensors contribute to the response to N starvation in *Chlorella* sp. by transducing extracellular stress signals to the cell that promote neutral lipid synthesis. The Ca^2+^ sensor calmodulin (CaM) senses changes in Ca^2+^ levels and then regulates proteins to produce the appropriate response^18^. The finding that RNAi-mediated down-regulation of the chloroplast-localized Ca^2+^ sensor (CAS) protein in *Chlamydomonas reinhardtii* strongly inhibits CEF, and is rescued by increasing the extracellular Ca^2+^ concentration, suggests that CEF is a Ca^2+^-dependent process^19^.

In this study, we aimed to identify the source of ATP during neutral lipid biosynthesis in green algae under N starvation, using both *C. sorokiniana* C3, an oil-producing microalga isolated from the wild, and the model green alga, *Chlamydomonas reinhardtii*. We found that the rate of CEF around PS I increased under N starvation to compensate for the reduced rates of photophosphorylation and drive ATP production for neutral lipid biosynthesis. Furthermore, we found that the *Chlamydomonas* mutant strain *pgrl1*, which exhibits restricted CEF, had significantly reduced neutral lipid accumulation under N starvation. In addition, the increase in cytosolic Ca^2+^ levels under N starvation served as a Ca^2+^ signal that was transmitted through the CaM and/or CAS-mediated pathway to increase CEF. Based on these findings, we propose a working model for enhanced ATP production for lipid biosynthesis during N starvation that involves Ca^2+^-regulated CEF.

## Results

### CEF around PS I is the main source of ATP for neutral lipid biosynthesis

By microscopy observation, 18S rDNA sequencing, and BLAST analysis, we isolated and identified a *Chlorella* strain named *C. sorokiniana* C3 (Fig. S1).

**Figure 1:**
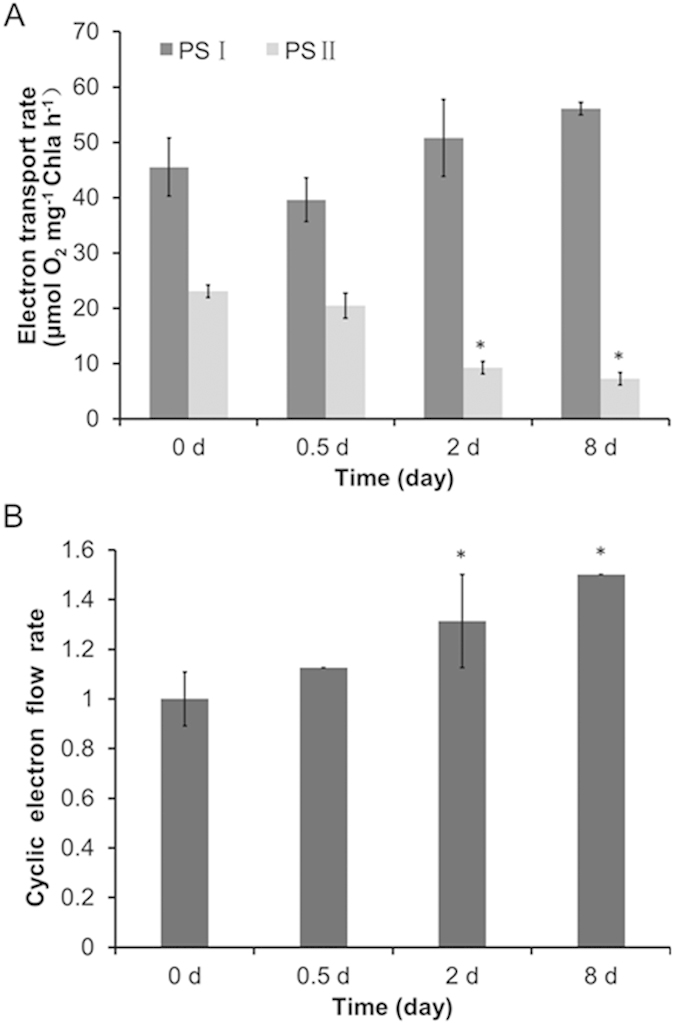
Electron transport rates in *Chlorella sorokiniana* C3 during oil droplet formation. (**A**) Electron transport rates via PS I and PS II. (**B**) CEF rates around PS I only, control (0 d) values were set to 1 for easy comparison. All data points in the current and following figures represent the means and SD of five biological replicates (*t* test, *P* < 0.05). The significance of the differences between the control (0 d) and other test values was tested using a one-way ANOVA. *P < 0.05; **P < 0.01).

CEF around PS I, or cyclic photophosphorylation, drives the production of ATP^20^. As shown in our previous study^16^, the key stages of N-induced oil droplet formation in *Chlorella* are as follows: day 0, the control stage (Cs); day 0–0.5, the pre-oil droplet formation stage (PDFs); day 0.5–2, the oil droplet formation stage (ODFs); and day 2–8, the late-oil droplet formation stage (LDFs). We proposed a role for CEF in ATP supply. To test whether CEF-mediated photophosphorylation supplies ATP for neutral lipid synthesis in *C. sorokiniana* C3 under N starvation, we measured the electron transport rate in the photosynthetic chain at the four key stages of oil droplet formation. As shown in Fig. 1A, the electron transport rate of PS II decreased continuously upon N- treatment, indicating decreased oxygenic photosynthesis driven by LEF^16^. By contrast, the electron transport rate via PS I, including both LEF and CEF, declined slightly at 0.5 d (the PDFs), and then slowly increased until 8 d (the ODFs) (Fig. 1A). Together, these results indicate that the rate of CEF around PS I increased. We confirmed this finding by direct CEF rate measurements (Fig. 1B).

**Figure 2:**
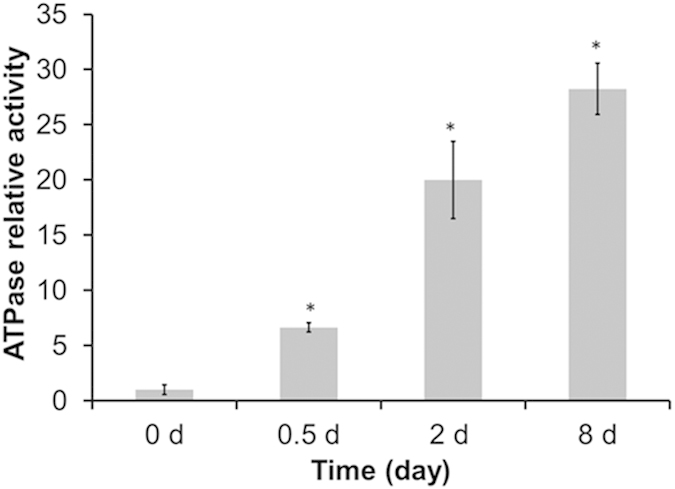
Analysis of ATP synthetase activity in the thylakoid membrane of *Chlorella sorokiniana* C3 during oil droplet formation. Control (0 d) values were set to 1 for easy comparison.

We then monitored the activity of ATP synthetase, the final producer of ATP, to further determine the main source of ATP for neutral lipid synthesis in *C. sorokiniana* C3 under N starvation. As shown in Fig. 2, the activity of ATP synthetase in the thylakoid membrane increased significantly during OD formation under N starvation. As the decreased LEF rate resulting from N- treatment (Fig. 1A) inhibited the generation of a transmembrane proton gradient that could drive ATP synthesis in the chloroplast, the increased ATP synthetase activity was driven by the transmembrane proton gradient generated by the CEF around PS I (Fig. 1B). This finding indicates that CEF is the main provider of ATP for neutral lipid synthesis in *C. sorokiniana* C3 under N starvation.

### Deficiency of CEF results in decreased N starvation-induced neutral lipid synthesis

**Figure 3:**
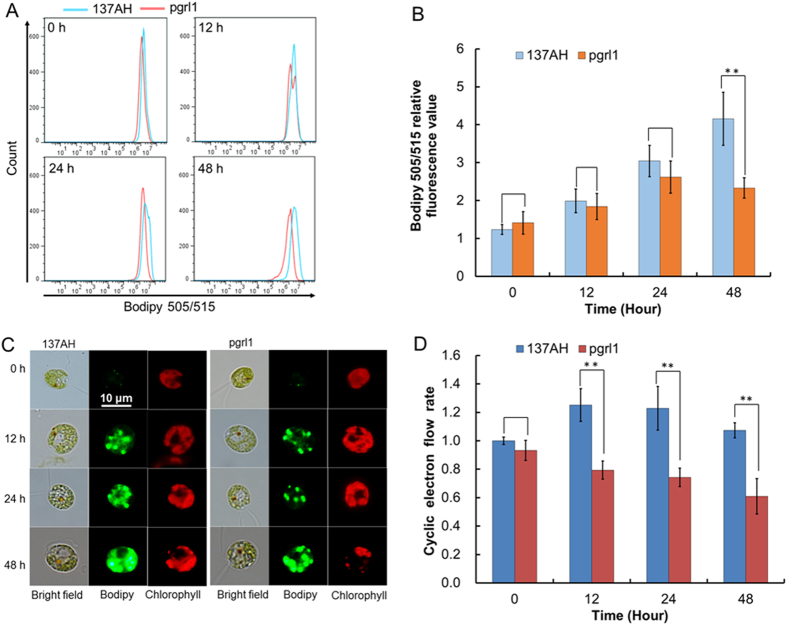
Lipid accumulation and CEF rates around PS I of *Chlamydomonas reinhardtii* 137AH and *pgrl1* under N starvation. Lipid accumulation was analyzed by FCM (**A**) and CLSM (**C**). (**B**) Bodipy relative fluorescence value analyzed by FCM. (**D**) CEF rates around PS I only. Control (0 d of *C. reinhardtii* 137AH) values were set to 1 for easy comparison.

To confirm the contribution of CEF to neutral lipid synthesis under N starvation in the model green alga, we monitored neutral lipid synthesis in a knock-out *C. reinhardtii* mutant (*pgrl1*) deficient in PGRL1-mediated CEF using both FCM (Fig. 3A,B) and fluorescence microscopy (Fig. 3C). Since the N starvation-mediated induction of oil droplet formation in *C. reinhardtii* was much faster than in *Chlorella*, we set induction times of 12, 24, and 48 h. Compared with its wild-type progenitor, *C. reinhardtii* strain 137AH, neutral lipid synthesis in the CEF deficient mutant strain *pgrl1* was significantly inhibited under N starvation (Fig. 3A–C). This was accompanied by an increase in CEF in the 137AH strain, but a decrease in CEF in the mutant strain (Fig. 3D). The significant difference in both CEF rate and neutral lipid accumulation between the wild-type and mutant strains indicated that deficiency of CEF blocked neutral lipid synthesis under N starvation and confirmed that CEF around PS I supplied ATP for neutral lipid synthesis.

**Figure 4:**
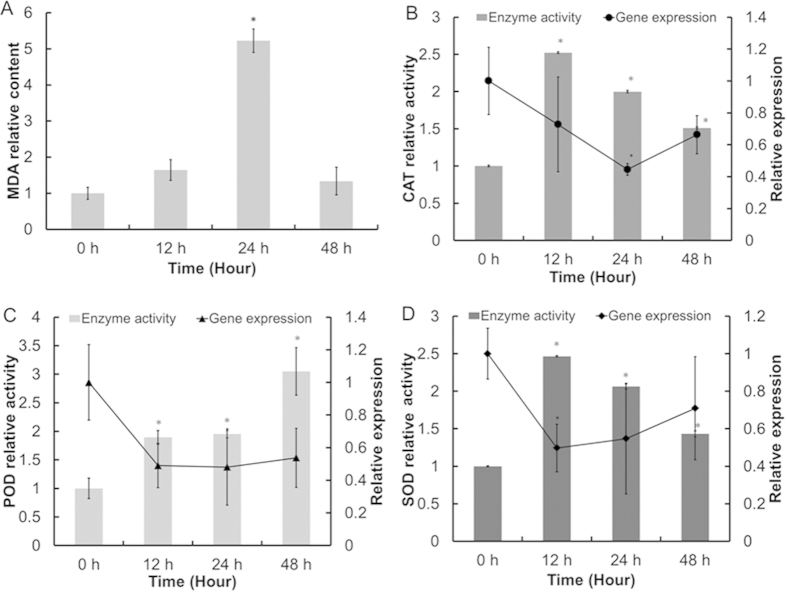
Lipid peroxidation level, antioxidant enzymes activities, and gene transcript levels of *Chlamydomonas reinhardtii* 137AH during oil droplet formation. (**A**) MDA content. (**B**–**D**) CAT, POD, and SOD activities and transcript levels. Control (0 d) values were set to 1 for easy comparison.

Similar to our observations in *C. sorokiniana* C3, N starvation also increased oxidative stress in *C. reinhardtii* 137AH cells, as shown by the increase in Malondialdehyde (MDA) levels (Fig. 4A). This finding indicates that N starvation-induced oxidative stress caused membrane system damage, which further induced neutral lipid synthesis in cells. SOD, CAT, and POD activity increased at different time points after N- treatment, which would reduce ROS to finally reduce MDA levels in cells (Fig. 4B–D). We next analyzed the transcript levels of genes encoding the three antioxidant enzymes in *C. reinhardtii* 137AH using real-time fluorescence quantitative PCR. Whereas the activities of SOD, CAT, and POD increased after N- treatment, the levels of the corresponding gene transcripts decreased, indicating that the enzymatic activities of these enzymes are not regulated at the transcriptional level, but possibly are regulated at the translational level. This possibility requires further testing.

### Ca^2+^ signal transduction regulates CEF during N starvation

**Figure 5:**
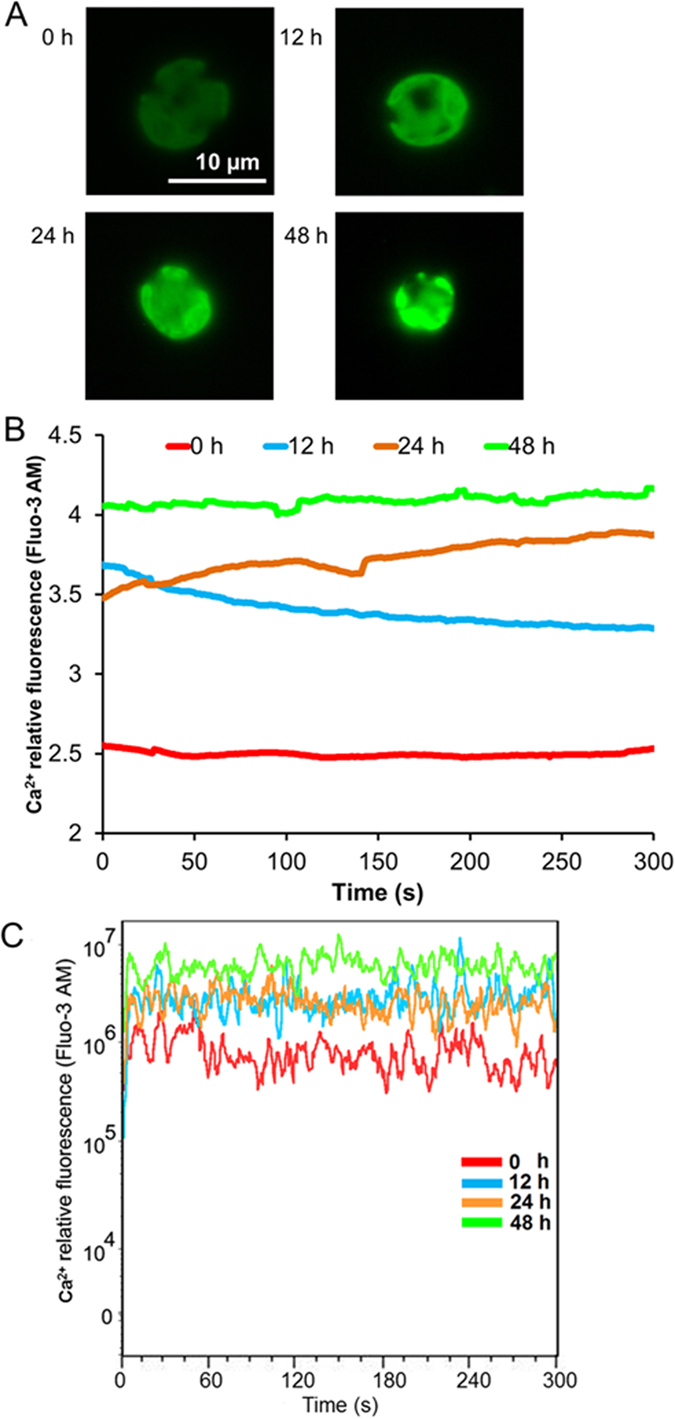
Analysis of cytosolic Ca^2+^ levels in *Chlamydomonas reinhardtii* 137AH under N starvation. Fluo-3 AM fluorescence in a single cell (**A**,**B**) or large number of cells (**C**) was detected after 0 h, 12 h, 24 h, and 48 h of N starvation. (**A**) Fluorescence image of cells. (**B**) Fluo-3 AM fluorescence in a single cell. The data points and figures represent the means of five replicates per sample. (**C**) Fluo-3 AM fluorescence in a large number of cells. The data points at each second represent the means of 2 × 10^3^–3 × 10^3^ cells in five replicate studies.

We then monitored the fluorescence of Fluo-3 AM, a Ca^2+^-sensitive fluorescent dye, in single *reinhardtii* 137AH cells subjected to N starvation. We found that N starvation caused a significant increase in fluorescence intensity, and that the fluorescence intensity was increased when the treatment was prolonged (i.e., 12–48 h; Fig. 5A,B). Based on our analysis of *C. reinhardtii* 137AH cells (>10^4^), we found that there was a significant increase in fluorescence intensity in cells subjected to the N- treatment for a prolonged period compared to cells that were cultured under normal growth conditions (N+; Fig. 5C), suggesting that the cytosolic Ca^2+^ level increased and formed a Ca^2+^ signal in response to N starvation.

**Figure 6:**
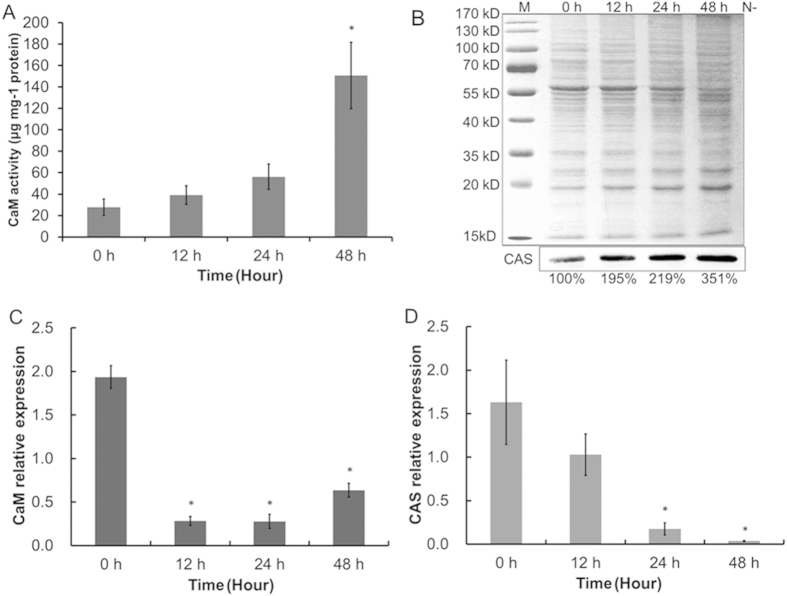
Protein level and gene transcript levels of CaM and CAS in *Chlamydomonas reinhardtii* 137AH under N starvation. Protein level of CaM (**A**) and CAS (**B**) were detected using CaM active enzyme continuous reaction spectrophotometry and immunoblotting, and control (0 d of *C. reinhardtii* 137AH) value of CAS activity was set to 100% for easy comparison. Gene transcript levels of CaM (**C**) and CAS (**D**) were detected using real-time RT-PCR after 0 h, 12 h, 24 h, and 48 h of N starvation. B, M, protein marker.

Proteins that bind to Ca^2+^, including CaM and the chloroplast-localized Ca^2+^ sensor protein (CAS), regulate the activity of proteins in response to changes in the Ca^2+^ signal to produce an appropriate response^19^,^21^. CAS regulates the ferredoxin plastoquinone reductase PGRL1, which is involved CEF around PS I^19^, in response to changes in the Ca^2+^ signal. Within 48 h of exposure to N starvation, CaM activity and CAS protein level in *C. reinhardtii* 137AH were enhanced 5.4 and 3.51 fold, respectively (Fig. 6A,B). This finding further suggests that the increase in cytosolic Ca^2+^ level transmitted Ca^2+^ signals to downstream pathways via interactions with calcium-binding proteins (CaM and CAS) to regulate the increased CEF rate, which in turn provides ATP for neutral lipid synthesis in *C. reinhardtii* 137AH in response to N starvation.

Interestingly, despite the large increase in CaM activity and CAS protein level in response to N starvation, the transcript levels of genes encoding CaM and CAS in *C. reinhardtii* 137AH were decreased to 32.8% and 2.3%, respectively, of their initial levels within 48 h of N- treatment (Fig. 6C,D). The discrepancy between protein activities and corresponding gene transcript levels indicates that, similar to the ROS scavenging enzymes (Fig. 4), neither CaM nor CAS is regulated at the transciriptional level.

## Discussion

Algae are adversely affected by several environmental factors, such as nutrient imbalances, radiation, salinity, and extreme temperatures, which have a negative effect on their survival and development^22^. Among these environmental factors, nutrient elements are considered to be the main factors limiting algal survival, growth, and productivity^23^. Limitation of N, one of the most important nutrient elements, reduces CO_2_ assimilation^24^, thereby increasing the accumulation of NADPH, and the excess reducing power causes oxidative stress. A shortage of electron acceptors (NADP+ or oxidized Fd) from PS I due to low CO_2_ fixation activity reduced the efficiency of PS II photochemistry driven by LEF^25^, which repressed ROS production by facilitating the consumption of excess reducing power and prevented the over-reduction of cells in a redox state^26^,^27^. Both photosynthesis and respiration were inhibited in *C. sorokiniana* C3 cells subjected to N starvation stress, as demonstrated by the reduced respiration rate, photosynthetic rate, photochemistry efficiency^16^, and LEF rate (Fig. 1). However, the decreased LEF rate resulted in a decrease in ATP production (Fig. 2) due to a reduction of the transmembrane proton gradient generated by LEF. The rates of CEF increased to compensate for the loss in ATP production (Fig. 1). Gao, *et al.*^28^ implied that the LEF was abolished in desiccated *Ulva* sp., whereas the cyclic PSI activity was significantly elevated, was still active at severe levels of desiccation and could be restored faster than PSII activity, concluding the PSI-driven CEF might provide desiccation tolerance and additional flexibility for the cell physiology of *Ulva* sp. under desiccation conditions. Furthermore, Gao, *et al.*^29^ also have reported that CEF around PSI was still active and increased significantly after inactivation of LEF following severe desiccation of the intertidal macroalgae *Porphyra haitanensis*, suggesting CEF in *P. yezoensis* played a significant physiological role during desiccation and re-hydration^30^. Joliot and Joliot^31^ showed that inhibition of LEF in the absence of CO_2_ stimulated CEF and also induced the formation of a large proton gradient. Similarly, in *C. reinhardtii* mutants devoid of Rubisco or ATPase, where the reducing power cannot be used for carbon fixation, CEF was stimulated, and could operate under aerobic conditions to support a simple competition model such that the excess reducing power was recycled to match the demand for ATP^14^.

Compounds or metabolites with special features are synthetized in cells in response to N limitation or starvation. For example, lipids/triacylglycerols (TAGs) often accumulate in microalgal cells subjected to N limitation or starvation^2^,^9^,^12^,^16^, which is the technical basis of algae-based biodiesel production. Whereas N starvation-induced lipid biosynthesis itself requires ATP, both the photophosphorylation operated by LEF and respiratory oxidative phosphorylation dropped significantly and failed to supply sufficient ATP^16^. Our results suggest that the increased CEF rate, which generates a proton gradient across the thylakoid membrane, contributed to ATP production for TAG synthesis in *C. sorokiniana* C3 under N starvation (Figs 1 and 2). We further showed that an increase in CEF rate drives the production of ATP, which is needed for neutral lipid synthesis under N starvation (Fig. 3).

Ca^2+^ is a ubiquitous intracellular second messenger in signal transduction pathways conveying environmental stimuli in plants^32^. Specific changes in cytosolic Ca^2+^ levels occur when plants or microalgae are exposed to various environmental stresses, and Ca^2+^ signals transfer extracellular stimuli to cells to regulate the response to the stresses^33^. We previously suggested that, by transducing extracellular stress signals into the cell and regulating the Ca^2+^ signal in neutral lipid synthesis, Ca^2+^ signal transduction plays important roles in the response mechanism of *Chlorella* sp. C2 to N starvation^18^. In this study, we found that the cytosolic Ca^2+^ level in *C. reinhardtii* 137AH also increased and formed a Ca^2+^ signal during neutral lipid synthesis under N starvation (Fig. 5). CaM and chloroplast-localized CAS further transmitted the Ca^2+^ signal into the chloroplast to regulate CEF and produce an appropriate response (Fig. 6). Furthermore, Terashima, *et al.*^19^ showed the Ca^2+^-dependent regulation of CEF via chloroplast-localized CAS. Thus, Ca^2+^ signal transduction contributes to neutral lipid synthesis by regulating CEF via CaM and CAS.

In accordance with a previous study^16^, we showed that N starvation ultimately results in oxidative stress in *Chlamydomonas* (Fig. 4). During N starvation-induced oil droplet formation, the absorbed light energy could not be consumed effectively by down-regulated LEF (Fig. 1) and resulted in the production of ROS in cells (Fig. 4). Furthermore, depressed NADPH consumption in the following carbon fixation also causes excess reducing power to accumulate, which in turn leads to oxidative stress^25^. The stimulated CEF (Figs 1 and 5) protects photosystems from oxidative stress, possibly by (1) directly dissipating light energy to drive the electron transfer and the formation of the proton gradient for ATP synthesis (which could then energize the lipid biosynthesis pathway) and (2) increasing ATP production to reduce the ATP/NADPH ratio. Therefore, as an important electron transfer pathway, the stimulated Ca^2+^-mediated CEF supplemented the depressed LEF in photosystems under N starvation, suggesting the key role in protecting and sustaining the operation of photosystems.

**Figure 7:**
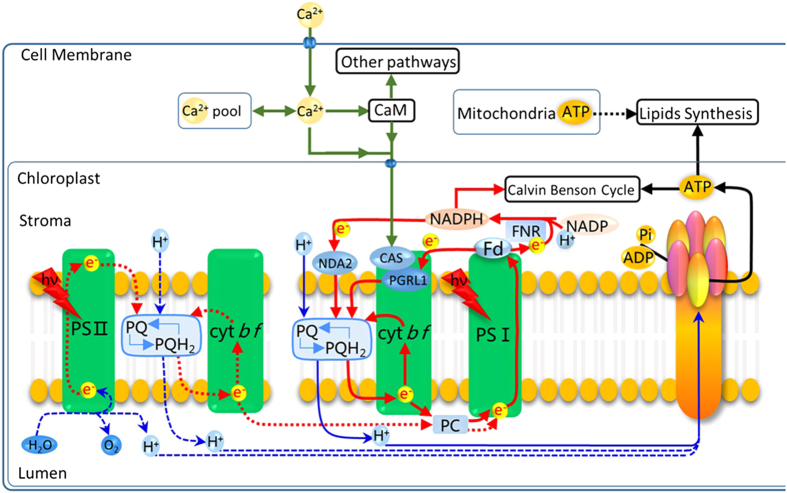
Regulation of neutral lipid synthesis in microalgae subjected to N starvation. When microalgae are exposed to N starvation, photophosphorylation and respiratory oxidative phosphorylation, the main sources of ATP are inhibited and there is a shortage of ATP. The resulting environmental stimuli are recognized by membrane sensors and the sensors activate the Ca^2+^ channels in the plasmalemma and the membranes of intracellular calcium stores through a series of phosphorylation reactions, all of which result in rapid rises in Ca^2+^ levels in the cytoplasm. The Ca^2+^ signals are further transmitted to the chloroplast via interactions with CaM and/or chloroplast-localized CAS to induce the increase of PGRL1-mediated CEF rate around PS I to drive the formation of a transmembrane proton gradient and then activate ATP synthetase to produce more ATP for neutral lipid synthesis, re-equilibrate the ATP/NADPH balance, and recycle excess light energy in photosystems to prevent ROS production.

Thus, as an important operating mode of the photosynthetic chain in photosystems, CEF plays many regulatory roles in cellular physiological processes besides ATP production. Based on the data in our previous^16^,^21^ and present study, we propose a scenario (Fig. 7) in which a series of mechanisms is sequentially triggered in the oil-producing green algae in response to an increased ATP demand to produce neutral lipids and re-equilibrate the ATP/NADPH imbalance resulting from the inhibition of photophosphorylation driven by LEF and respiratory oxidative phosphorylation (Fig. 3). When algae are cultured under N-sufficient conditions, photophosphorylation driven by the photosynthetic chain in photosystems and respiratory oxidative phosphorylation provide most of the ATP for cellular processes and maintain the ATP/NADPH balance^13^,^14^,^15^. However, when algae are exposed to N starvation, these mechanisms fail to dissipate the excess light energy and NADPH and to compensate for the ATP deficit, as photophosphorylation driven by LEF and respiratory oxidative phosphorylation are inhibited^16^. As a consequence, the environmental stimuli are recognized by membrane sensors and activate Ca^2+^ channels in the cell membrane (plasmalemma) and membranes of the intracellular calcium stores through a series of phosphorylation reactions^18^, all of which rapidly increase Ca^2+^ levels in the cytoplasm (Fig. 5). The Ca^2+^ signal is transmitted to the chloroplast via interactions with CaM and/or chloroplast-localized CAS to increase the PGRL1-mediated CEF rate around PS I to drive the formation of a transmembrane proton gradient and then activate ATP synthetase to produce more ATP for neutral lipid synthesis, re-equilibrate the ATP/NADPH balance, and recycle excess light energy in photosystems to prevent ROS production.

In summary, to compensate for the reduction in ATP synthesis during N starvation, microalgae increase the rate of PGRL1-mediated CEF around PS I. This mechanism is regulated by a Ca^2+^ signal transduction pathway that involves CaM and/or CAS. Simultaneously, CEF also re-equilibrates the ATP/NADPH balance and recycles excess light energy in photosystems to prevent ROS production, which played the key role in protecting photosystems.

## Materials and Methods

### Strains

*Chlorella* strains were separated and provided by Professor Xudong Xu (Key Laboratory of Algal Biology, Institute of Hydrobiology, Chinese Academy of Sciences). *Chlorella* strains were identified by microscopy observation and 18S rDNA sequencing and BLAST analysis were performed as described by Xu and Hu^34^. The identified *C. sorokiniana* C3 was used in this study.

The *Chlamydomonas reinhardtii* wild-type strain 137AH (*mt- nit1 nit2*), and a knock-out *C. reinhardtii* mutant (*pgrl1*) deficient in PGRL1-mediated CEF were provided by Professor Gilles Peltier (CEA, Institut de Biologie Environnementale et de Biotechnologie, France)^35^.

### Growth conditions and N- treatment

The N-sufficient medium (N+) used for *C. sorokiniana* C3 was full-strength BG11 medium^36^. The N-deficient medium (N−) was BG11 without NaNO_3_. *C. sorokiniana* C3 was cultured and subjected to N treatment as previously described^16^,^18^,^37^. *C. sorokiniana* C3 in the exponential phase was inoculated into a 1 liter Erlenmeyer flask containing 500 ml BG11 medium at 25 °C with continuous illumination of 70 μmol m^−2^ s^−1^ and continuously bubbled with filtered air, the initial OD_700_ is 0.05. For N- treatment, cells were harvested by centrifugation at 3,000 g for 3 min at 25 °C when they reached the midlogarithmic growth phase (OD_700_ approximately 0.8), and were then washed and resuspended in N- medium to OD_700_ 0.3.

The *pgrl1* knockout mutant of *C. reinhardtii* and *C. reinhardtii* wild-type strain 137AH, the progenitor of *pgrl1* knockout mutant, were grown as described by Tolleter, *et al.*^38^ with minor modifications. Cells in the exponential phase were inoculated into a 1 liter Erlenmeyer flask containing 500 ml TAP medium at 25 °C with continuous illumination of 40 μmol m^−2^ s^−1^ and continuously bubbled with filtered air, the initial OD_700_ is 0.05. The N- medium used for *C. reinhardtii* was TAP without NH_4_Cl. N- treatment of *C. reinhardtii* was the same as for *C. sorokiniana* C3.

### Lipid Analysis

#### Confocal Laser Scanning Microscopy (CLSM) analysis

Microscopy analysis of cells stained with Bodipy 505/515 (Sigma Aldrich, USA) was carried out using a confocal laser scanning microscope (Zeiss LSM 710 NLO). Non-fluorescent protoplast structures were visualized using the manufacturer’s recommended filter settings. Specific experimental processes were previously described^16^,^21^,^37^. A lipophilic fluorescent dye, Bodipy 505/515 (4,4-difluoro-1,3,5,7-tetramethyl-4-bora-3a, 4a-diaza-sindacene), was used to stain the intracellular oil-containing organelles, known as lipid bodies, with a final labeling concentration of 1 μM and 0.1% DMSO (v/v), according to Cooper, *et al.*^39^. Bodipy fluorescence (green) was excited with an argon laser (488 nm) and detected at 505–515 nm. Autofluorescence (red) of algal chloroplasts was detected simultaneously at 650–700 nm.

#### Flow Cytometry (FCM) analysis

Samples stained with Bodipy 505/515 were analyzed on a board using a FACSAria Flow Cytometer (Becton Dickinson, San Jose, CA, USA) equipped with a laser emitting at 488 nm and an optical filter FL1 (530/30 nm)^16^,^18^,^37^. The collected data were analyzed using FlowJo software (Tree Star, San Carlos, CA, USA).

### Electron transport rates measurement

Electron transport rates were estimated by measuring O_2_ consumption/evolution using a Clark-type electrode (Hansatech) at 20 °C as described by Wang, *et al.*^40^. The light intensity used was 500 μmol m^−2^ s^−1^ white light. Thylakoid membranes were adjusted to a chlorophyll content of 15 μg/mL for all measurements. The PS I reaction mixture contains 40 μM MV, 5 mM NH4Cl, 2 mM ascorbic acid, 0.1 mM 2,6-dichlorophenolindophenol (DCPIP), 2 mM NaN3, 40 μM 3-(3,4-dichlorophenyl)-1,1-dimethylurea (DCMU), 40 mM tricine (pH 7.5), and 100 mM Suc. The PS I activity was determined by measuring the electron transfer from DCIP via PS I to MV; one oxygen molecule is consumed for each electron transport event. The PS II reaction mixture contains 5 mM NH_4_Cl, 4 mM K3FeCN, 1 mM phenyl-p-benzoquinone, 40 mM tricine (pH 7.5), and 100 mM Suc. The electron transport rates of PS II were determined by measuring the electron transfer from H_2_O to phenyl-p-benzoquinone (BQ); one oxygen molecule is produced for every four electrons transported. The CEF rates around PS I reaction mixture contains 5 mM NH_4_Cl, 10 μM DCMU, 0. 5 mM NADPH, 5 μM ferredoxin, 10 mM NaCl, 5 mM MgCl_2_, 10 mM KCl, 0.25 mM KH_2_PO_4_, 2 mM ethylene diamine tetraacetic acid (EDTA), 1 mM MnCl_2_ and 50 mM 4-(2-Hydroxyethyl)-1-piperazineethanesulfonic acid (HEPES, pH 7.6). While PS II activity was blocked by addition of DCMU, the sustained steady-state electron transfer is attributed to cyclic electron flow around PS I. The CEF rates around PS I were determined by measuring the electron transfer from NADPH via PS I to O_2_^41^,^42^ in the presence of 5 μM ferredoxin (Sigma Aldrich, USA), 0.5 mM NADPH (Sigma Aldrich, USA), and 10 μM DCMU; one oxygen molecule is consumed for each electron transport event. O_2_ evolution/consumption was followed for 3 min, and the rate was calculated accordingly.

### ATP synthetase activity assays

ATP synthetase activity assays were performed using an ATP Synthetase Assay Kit (GENMED, USA). A 100 ml culture at OD_700_ = 1 (about 1.3 × 10^7^ cells ml^–1^) was harvested by centrifugation at 3,000 g for 3 min at room temperature, and the algal pellets were washed with 1 ml Reagent A in Kit, and centrifugation was repeated. The washed algal pellets were resuspended in pre-cooling 4 ml of Reagent B. The suspension was sonicated at 200 W for 30 min (3 s working time and 3 s interval in a cycle) in an ultrasonic cell disruptor at 4 °C, and the supernatant was collected by centrifugation at 1,600 g for 10 min at 4 °C. Then supernatant was centrifuged at 10,000 g for 60 min at 4 °C to collect sediment. The collected sediment was dissolved into 200 μl Reagent B and used for the activity assays of the ATP synthetase in thylakoid membrane according to the manufacturer’s instructions. Protein content was assayed using a BCA Protein Quantification Kit (TIANGEN, China). ATP synthetase activity (U mg^−1^ protein) in thylakoid membrane was related to the amount of protein in the chloroplast homogenate and defined as the amount of enzyme that caused per micromoles NADH oxidation per minute per milligram of protein at 37 °C and pH 8.0.

### Lipid peroxidation assessment and ROS scavenging enzyme activity assays

MDA level, CAT, POD and SOD activities were measured according to Shi *et al.* (2009) with some modifications as shown in Zhang *et al.* (2013). Cells (10^7^ cells ml^–1^) were harvested by centrifugation at 3,000 g for 3 min, and the cell pellet was washed and then resuspended with 0.2 M sodium phosphate buffer (pH 7.8, containing 4 mM EDTA-Na_2_, 0.4% PVP). The resuspended cells were homogenized at 4 °C and then centrifugated at 13,000 g for 30 min at 4 °C. The supernatants were used for MDA and enzyme activity analysis directly. Protein content was assayed using BCA Protein Quantification Kit (TIANGEN, China). MDA levels and CAT, POD, and SOD activities were then measured using an MDA Assay Kit (Beyotime, China), CAT Activity Assay Kit (Beyotime, China), POD Activity Assay Kit (Nanjing Bioengineering Institute, China), and SOD Activity Assay Kit (Beyotime, China), according to the manufacturer’s instructions. MDA level (nM mg^−1^ protein) was related to the amount of protein in the cell homogenate and expressed as nanomole of MDA per milligram of cell protein. CAT activity (U mg^−1^ protein) was defined as the amount of enzyme that caused per micromoles H_2_O_2_ reduction per second per milligram of cell protein at 37 °C. POD activity (U mg^−1^ protein) was defined as the amount of enzyme that catalyzed per milligram substrate per minute per milligram of cell protein at 37 °C. SOD activity (U mg^−1^ protein) was defined as the amount of enzyme that caused a 50% decrease of the SOD inhabitable NBT reduction per milligram of cell protein at 37 °C.

### Real-time RT-PCR analysis

Cells (10^7^ cells/ml) were harvested and resuspended in a 1.5 ml micro-tube containing 1 ml TRIZOL Reagent (Invitrogen, USA). After precipitation in 100% isopropanol and washing in 75% ethanol, the RNA pellet was suspended in a suitable volume of DEPC water according to the manufacturer’s instructions. RNA solutions were quantified using a NanoDrop 3.0.0 (Coleman Technologies Inc., USA). Aliquots were stored at −70 °C.

The transcriptional expression of genes encoding CAT, SOD, POD, CaM, and CAS was measured using real-time RT-PCR^43^. First strand synthesis was carried out using a PrimeScript RT Reagent Kit With gDNA Eraser according to the manufacturer’s instructions (#RR047A, TAKARA). To perform the gene expression analyses, specific primer sets were designed to produce 100 to 200 bp PCR products (Table S1). Quantitative real-time PCR was performed (three technical replicates on five biological replicates) using iTaq Universal SYBR Green Supermix (#172, Bio-Rad) and a Bio-Rad CFX96 Thermal Cycler (Bio-Rad, USA). Differences in expression were calculated according to the ‘delta–delta method’ (Pfaffl 2001), using 18S rRNA and CBLP as references.

### Fluorescence detection of cytosolic Ca^2+^

For fluorescence imaging of cytosolic Ca^2+^, the cells were loaded with a Ca^2+^-sensitive fluorescent dye, Fluo-3 AM, according to Chen, *et al.*^18^. Fluorescence images were obtained using the Ratio Fluorescence Imaging System (EasyRatioPro, PTI, USA), and changes in fluorescence were recorded in a single cell. The excitation and emission wavelengths were 488 nm and 525 nm, respectively. Fluo-3 AM fluorescence was continuously recorded for 5 min. Samples stained with Fluo-3 AM were also analyzed on a board using a FACSAria flow cytometer (Becton Dickinson, San Jose, CA, USA) equipped with a laser emitting at 488 nm and an optical filter FL1 (530/30 nm). Fluo-3 AM fluorescence of cells (>10^4^) was continuously recorded for 5 min. Data were analyzed using FlowJo software (Tree Star, San Carlos, CA, USA).

### CaM activity assays

CaM activity was measured using CaM active enzyme continuous reaction spectrophotometry, which is based on activation of a CaM-dependent cyclic nucleotide phosphodiesterase^44^. CaM activity assays were performed using a Plant CaM Active Enzyme Continuous Reaction Spectrophotometry Assay Kit (GENMED, USA). A 50 ml culture at OD_700_ = 1 (about 1.3 × 10^7^ cells ml^–1^) was harvested by centrifugation at 3,000 g for 3 min at room temperature, and the algal pellets were washed with 3 ml Reagent A in Kit, and centrifugation was repeated. The washed algal pellets were resuspended in pre-cooling 1 ml of Reagent B. The suspension was sonicated at 200 W for 30 min (3 s working time and 3 s interval in a cycle) in an ultrasonic cell disruptor at 4 °C. The suspension was boiled for 90 s and then cooled for 5 min in ice. Then supernatant was collected by centrifugation at 10,000 g for 5 min at 4 °C. The supernatants were used for CaM activity analysis directly according to the manufacturer’s instructions. Protein content was assayed using a BCA Protein Quantification Kit (TIANGEN, China). CaM activity (μg mg^−1^ protein) was related to the amount of protein in the cell homogenate and expressed as milligrams of CaM activity per milligram of cell protein.

### Immunoblot assays

Protein analysis and immunodetection were performed as previously described^45^. Antibody against CAS (against the peptide sequence ARADELDSTVESVVG^19^) was produced in rabbits. The densitometric quantitation of CAS activity was determined accordingly by using ImageJ (ver1.41, NIH)^46^ and calculated as a relative value of control.

### Statistical analyses

Each result shown is the mean of five biological replicates. Statistical analysis of the data was performed using the program SPSS-13 and significance was determined at 95% or 99% confidence intervals. *t* test was used to determine the means and SD of replicated studies. The significant differences between the control and test values were tested by using one-way ANOVA test, and differences were considered to be significant at *P* < 0.05 or *P* < 0.01.

## Additional Information

**How to cite this article**: Chen, H. *et al.* Ca^2+^-regulated cyclic electron flow supplies ATP for nitrogen starvation-induced lipid biosynthesis in green alga. *Sci. Rep.* **5**, 15117; doi: 10.1038/srep15117 (2015).

## Supplementary Material

Supporting Information

## References

[b1] Chisti Y. Biodiesel from microalgae. Biotechnology Advances 25, 294–306, 10.1016/j.biotechadv.2007.02.001 (2007). 17350212

[b2] Zhang X., Rong J., Chen H., He C. & Wang Q. Current status and outlook in the application of microalgae in biodiesel production and environmental protection. Frontiers in Energy Research 2, 10.3389/fenrg.2014.00032 (2014).

[b3] Bourgis F. *et al.* Comparative transcriptome and metabolite analysis of oil palm and date palm mesocarp that differ dramatically in carbon partitioning. Proceedings of the National Academy of Sciences of the United States of America 108, 12527–12532, 10.1073/pnas.1106502108 (2011). 21709233 PMC3145713

[b4] Troncoso-Ponce M. A. *et al.* Comparative deep transcriptional profiling of four developing oilseeds. Plant Journal 68, 1014–1027, 10.1111/j.1365-313X.2011.04751.x (2011). 21851431 PMC3507003

[b5] Chapman K. D. & Ohlrogge J. B. Compartmentation of Triacylglycerol Accumulation in Plants. Journal of Biological Chemistry 287, 2288–2294, 10.1074/jbc.R111.290072 (2012). 22090025 PMC3268389

[b6] Moellering E. R. & Benning C. RNA Interference Silencing of a Major Lipid Droplet Protein Affects Lipid Droplet Size in *Chlamydomonas reinhardtii*. Eukaryotic Cell 9, 97-106, 10.1128/Ec.00203-09 (2010). 19915074 PMC2805299

[b7] Hu B. *et al.* Development of an effective acidogenically digested swine manure-based algal system for improved wastewater treatment and biofuel and feed production. Applied Energy 107, 255–263, http://dx.doi.org/10.1016/j.apenergy.2013.02.033 (2013).

[b8] Chen H., Qiu T., Rong J., He C. & Wang Q. Microalgal biofuel revisited: An informatics-based analysis of developments to date and future prospects. Applied Energy 155, 585–598, http://dx.doi.org/10.1016/j.apenergy.2015.06.055 (2015).

[b9] Mata T. M., Martins A. A. & Caetano N. S. Microalgae for biodiesel production and other applications: A review. Renewable & Sustainable Energy Reviews 14, 217–232, 10.1016/j.rser.2009.07.020 (2010).

[b10] Rodolfi L. *et al.* Microalgae for Oil: Strain Selection, Induction of Lipid Synthesis and Outdoor Mass Cultivation in a Low-Cost Photobioreactor. Biotechnol Bioeng 102, 100–112 (2009). 18683258 10.1002/bit.22033

[b11] Li X. B., Benning C. & Kuo M. H. Rapid Triacylglycerol Turnover in Chlamydomonas reinhardtii Requires a Lipase with Broad Substrate Specificity. Eukaryotic Cell 11, 1451–1462, 10.1128/Ec.00268-12 (2012). 23042128 PMC3536278

[b12] Scott S. A. *et al.* Biodiesel from algae: challenges and prospects. Current Opinion In Biotechnology 21, 277–286, 10.1016/j.copbio.2010.03.005 (2010). 20399634

[b13] Alric J. Cyclic electron flow around photosystem I in unicellular green algae. Photosynth Res 106, 47–56, 10.1007/s11120-010-9566-4 (2010). 20532629

[b14] Alric J., Lavergne J. & Rappaport F. Redox and ATP control of photosynthetic cyclic electron flow in Chlamydomonas reinhardtii (I) aerobic conditions. Biochimica Et Biophysica Acta-Bioenergetics 1797, 44–51, 10.1016/j.bbabio.2009.07.009 (2010). 19651104

[b15] Munekaga Y. *et al.* Cyclic electron flow around photosystem I is essential for photosynthesis. Nature 429, 579–582, 10.1038/Nature02598 (2004). 15175756

[b16] Zhang Y. M., Chen H., He C. L. & Wang Q. Nitrogen Starvation Induced Oxidative Stress in an Oil-Producing Green Alga Chlorella sorokiniana C3. PloS one 8, ARTN e69225 10.1371/journal.pone.0069225 (2013). PMC3712941 23874918

[b17] McAinsh M. R. & Pittman J. K. Shaping the calcium signature. New Phytologist 181, 275–294, 10.1111/j.1469-8137.2008.02682.x (2009). 19121028

[b18] Chen Z. *et al.* Phosphoproteomic Analysis Provides Novel Insights into Stress Responses in Phaeodactylum tricornutum, a Model Diatom. Journal of Proteome Research 13, 2511–2523, 10.1021/Pr401290u (2014). 24712722

[b19] Terashima M. *et al.* Calcium-dependent regulation of cyclic photosynthetic electron transfer by a CAS, ANR1, and PGRL1 complex. Proceedings of the National Academy of Sciences of the United States of America 109, 17717–17722, 10.1073/pnas.1207118109 (2012). 23045639 PMC3491457

[b20] Arnon D. I. Conversion of Light into Chemical Energy in Photosynthesis. Nature 184, 10–21 (1959). 13794394 10.1038/184010a0

[b21] Chen H., Zhang Y. M., He C. L. & Wang Q. Ca2+ Signal Transduction Related to Neutral Lipid Synthesis in an Oil-Producing Green Alga Chlorella sp C2. Plant Cell Physiol 55, 634–644, 10.1093/Pcp/Pcu015 (2014). 24449653

[b22] Fu W. Q. *et al.* Effects of abiotic stressors on lutein production in the green microalga Dunaliella salina. Microbial Cell Factories 13, Artn 3 10.1186/1475-2859-13-3 (2014). PMC3893366 24397433

[b23] Guschina I. A. & Harwood J. L. Lipids and lipid metabolism in eukaryotic algae. Progress in Lipid Research 45, 160–186, 10.1016/j.plipres.2006.01.001 (2006). 16492482

[b24] Schmollinger S. *et al.* Nitrogen-Sparing Mechanisms in Chlamydomonas Affect the Transcriptome, the Proteome, and Photosynthetic Metabolism. Plant Cell 26, 1410–1435, 10.1105/tpc.113.122523 (2014). 24748044 PMC4036562

[b25] Shikanai T. Cyclic electron transport around photosystem I: Genetic approaches. Annual Review of Plant Biology 58, 199–217, 10.1146/annurev.arplant.58.091406.110525 (2007). 17201689

[b26] Li X. B. *et al.* A Galactoglycerolipid Lipase Is Required for Triacylglycerol Accumulation and Survival Following Nitrogen Deprivation in Chlamydomonas reinhardtii. Plant Cell 24, 4670–4686, 10.1105/tpc.112.105106 (2012). 23161887 PMC3531859

[b27] Gonzalez-Ballester D. *et al.* RNA-Seq Analysis of Sulfur-Deprived Chlamydomonas Cells Reveals Aspects of Acclimation Critical for Cell Survival. Plant Cell 22, 2058–2084, 10.1105/tpc.109.071167 (2010). 20587772 PMC2910963

[b28] Gao S. *et al.* PSI-Driven Cyclic Electron Flow Allows Intertidal Macro-Algae Ulva sp (Chlorophyta) to Survive in Desiccated Conditions. Plant Cell Physiol 52, 885–893, 10.1093/pcp/pcr038 (2011). 21471121

[b29] Gao S. *et al.* The physiological links of the increased photosystem II activity in moderately desiccated Porphyra haitanensis (Bangiales, Rhodophyta) to the cyclic electron flow during desiccation and re-hydration. Photosynth Res 116, 45–54, 10.1007/s11120-013-9892-4 (2013). 23896795

[b30] Gao S. & Wang G. C. The enhancement of cyclic electron flow around photosystem I improves the recovery of severely desiccated Porphyra yezoensis (Bangiales, Rhodophyta). J Exp Bot 63, 4349–4358, 10.1093/jxb/ers082 (2012). 22438301

[b31] Joliot P. & Joliot A. Cyclic electron flow in C3 plants. Biochimica Et Biophysica Acta-Bioenergetics 1757, 362–368, 10.1016/j.bbabio.2006.02.018 (2006). 16762315

[b32] Sun Q. P., Guo Y., Sun Y., Sun D. Y. & Wang X. J. Influx of extracellular Ca^2+^ involved in jasmonic-acid-induced elevation of [Ca^2+^](cyt) and JR1 expression in *Arabidopsis thaliana*. Journal of Plant Research 119, 343–350, 10.1007/s10265-006-0279-x (2006). 16708291

[b33] Chinnusamy V., Schumaker K. & Zhu J. K. Molecular genetic perspectives on cross-talk and specificity in abiotic stress signalling in plants. J Exp Bot 55, 225–236, 10.1093/Jxb/Erh005 (2004). 14673035

[b34] Xu J. & Hu H. H. Screening high oleaginous Chlorella strains from different climate zones. Bioresource Technology 144, 637–643, 10.1016/j.biortech.2013.07.029 (2013). 23899578

[b35] Dang K. V. *et al.* Combined Increases in Mitochondrial Cooperation and Oxygen Photoreduction Compensate for Deficiency in Cyclic Electron Flow in Chlamydomonas reinhardtii. Plant Cell 26, 3036–3050, 10.1105/tpc.114.126375 (2014). 24989042 PMC4145130

[b36] Stanier R. Y., Kunisawa R., Mandel M. & Cohen-Bazire G. Purification and properties of unicellular blue-green algae (order *Chroococcales*). Bacteriological Reviews 35, 171–205 (1971). 4998365 10.1128/br.35.2.171-205.1971 PMC378380

[b37] Zhang X. *et al.* Evaluation of an Oil-Producing Green Alga Chlorella sp C2 for Biological DeNO(x) of Industrial Flue Gases. Environmental Science & Technology 48, 10497–10504, 10.1021/Es5013824 (2014). 25105531

[b38] Tolleter D. *et al.* Control of Hydrogen Photoproduction by the Proton Gradient Generated by Cyclic Electron Flow in Chlamydomonas reinhardtii. Plant Cell 23, 2619–2630, 10.1105/tpc.111.086876 (2011). 21764992 PMC3226202

[b39] Cooper M. S., Hardin W. R., Petersen T. W. & Cattolico R. A. Visualizing “green oil” in live algal cells. Journal of Bioscience and Bioengineering 109, 198–201, 10.1016/j.jbiosc.2009.08.004 (2010). 20129108

[b40] Wang Q. *et al.* The high light-inducible polypeptides stabilize trimeric photosystem I complex under high light conditions in Synechocystis PCC 6803. Plant Physiology 147, 1239–1250, 10.1104/pp.108.121087 (2008). 18502976 PMC2442545

[b41] Munekage Y. *et al.* PGR5 is involved in cyclic electron flow around photosystem I and is essential for photoprotection in Arabidopsis. Cell 110, 361–371, 10.1016/S0092-8674(02)00867-X (2002). 12176323

[b42] Okegawa Y., Kagawa Y., Kobayashi Y. & Shikanai T. Characterization of factors affecting the activity of photosystem I cyclic electron transport in chloroplasts. Plant Cell Physiol 49, 825–834, 10.1093/Pcp/Pcn055 (2008). 18388110

[b43] Gibson U. E. M., Heid C. A. & Williams P. M. A novel method for real time quantitative RT PCR. Genome Research 6, 995–1001, 10.1101/Gr.6.10.995 (1996). 8908519

[b44] Mac Neil S., Walker S. W., Senior H. J., Bleehen S. S. & Tomlinson S. Effects of extracellular calmodulin and calmodulin antagonists on B 16 melanoma cell growth. Journal of investigative dermatology 83, 15–19 (1984). 6736671 10.1111/1523-1747.ep12261637

[b45] Hu J. L., Deng X., Shao N., Wang G. H. & Huang K. Y. Rapid construction and screening of artificial microRNA systems in Chlamydomonas reinhardtii. Plant Journal 79, 1052–1064, 10.1111/Tpj.12606 (2014). 24974733

[b46] Tsihlis N. D. *et al.* Isopropylamine NONOate (IPA/NO) moderates neointimal hyperplasia following vascular injury. J Vasc Surg 51, 1248–1259, 10.1016/j.jvs.2009.12.028 (2010). 20223627 PMC2860688

