# Supplementary material for: Ca2+-regulated cyclic electron flow supplies ATP for nitrogen starvation-induced lipid biosynthesis in green alga
Source: Sci Rep. 2015 Oct 9;5:15117. doi: 10.1038/srep15117 (PMC4598854; doi:10.1038/srep15117)
Supplement: Supporting Information [file srep15117-s1.doc]

**Ca^2+^-regulated cyclic electron flow supplies ATP for nitrogen starvation-induced lipid biosynthesis in green alga**

Hui Chen, Jinlu Hu, Yaqin Qiao, Weixian Chen, Junfeng Rong, Yunming Zhang, Chenliu He, Qiang Wang

**Table S1.** Specific primers for gene quantitative real-time PCR analysis

| Gene | Primer sequence (5`-3`) | *C. reinhardtii* database matches [Pytozome locus name or GenBank Accession Number*] |
| --- | --- | --- |
| CAT1 | | CCGCGAGACGTATGTCAAGT | | --- | | GTCTTGGTCACGTCCAGAGG | | Cre09.g417150 |
| CAT2 | | TGTTCATGACGCCCTCAGAC | | --- | | GCAAAGGTCTGCAGGAAAGC | | Cre01.g045700 |
| Fe-SOD | | CCTACGCTCTGGATGCTCTG | | --- | | AGCGACCTGCTTGTTCATGT | | Cre10.g436050 |
| Mn-SOD1 | | AGGATGTCGCCACTGTCATC | | --- | | ACTTGGCCTTCATCTCGTCC | | Cre02.g096150 |
| Mn-SOD2 | | GCTACAACAAGGCCACACAC | | --- | | ACGTTCTGCCAGTTGACGAT | | Cre13.g605150 |
| Mn-SOD3 | | GGCTTACCACTGGCTTACGA | | --- | | GACAATCTCCGACAGCGACA | | Cre16.g676150 |
| Mn-SOD4 | | CTCATGGGGGTGGTGCTCAA | | --- | | GTGTAGTCCGGCTTCTTGGG | | Cre12.g490300 |
| Mn-SOD5 | | CTACCTCCAGTACCAGGCCA | | --- | | GATGTTGTAGCTGAGGTCCCC | | GU134345.1* |
| 2-cys peroxiredoxin | | TGGGTGAACTGGCTGTCAAT | | --- | | CCACCGAGATCACTGCCTTC | | Cre02.g114600 |
| 2-cys peroxiredoxin, chloroplastic | | GGTTCTGGGTGTGTCTGTGG | | --- | | GCCCTCCTTGTCGATGATGAA | | Cre06.g257601 |
| Ascorbate peroxidase | | CAACTGGCTCGAGTTCGACA | | --- | | CATAGGGGCGGAACTGATCG | | Cre02.g087700 |
| Glutathione peroxidase | | CTGATGCGTATTGTTGTCGTTC | | --- | | AGCTACCCGGTCCTTAGAAGAG | | Cre10.g440850 |
| Glutathione peroxidase5 | | GCTAGCAAGTGCGGCTTTAC | | --- | | AAATTGCGCTGGCAGAACTC | | Cre10.g458450 |
| Glutathione peroxidase3 | | ACGACCTCGGAGTTCTACCA | | --- | | GCTTGCCGTACTTGTCTTGC | | Cre03.g197750 |
| Glutathione peroxidase1 | TGACCATCGTCGCTTTCC  CACGTCCACCTTGTCCATC | Cre02.g078300 |
| L-ascorbate peroxidase2 | | CAAGACGAAGACTGCTGTGG | | --- | | CGTACTGGATGGAGGCGTTC | | Cre06.g285150 |
| L-ascorbate peroxidase, heme-containing | | GTCGAAGGATGAGGCCAAGAG | | --- | | GCTGGTGAACAGTGAGTAGCC | | g10003 |
| Peroxiredoxin3 | | CTGTTCGGAGGCAAGAAGGT | | --- | | ATAGGCAGGCAATGGTGTCC | | Cre10.g449550 |
| Peroxiredoxin5 | | ATGCTCGCCAACATCTTCCA | | --- | | TTTACAGAGCTCGTCCGTGG | | Cre01.g014350 |
| Peroxiredoxin4 | | GGACGTCTTCAAGGGCAAGA | | --- | | CCACAGTCACGCACAATACC | | Cre02.g080900 |
| Thioredoxin dependent peroxidase | | CACCGACGAGAACTCCATCC | | --- | | TTGAAGGCCATGACGCACTT | | Cre10.g422300 |
| 18S rRNA | | GCCTAGTAAGCGCGAGTCAT | | --- | | AGCCAAGCTCAATCCGAACA | | M32703* |
| CBLP | GCTGTGGGACCTGGCTGA  CCTTCTTGCTGGTGATGTTG | g6364 |
| CaM | AACGGCACCATTGATTTCC  TCATCTCATCGACCTCCTCC | NW_001843987.1 |
| CAS | TCAACTCGGGCACCAAGG  CGCCCACCACCACATACAC | AB127959.1 |

*Pytozome locus name database is available under <http://www.phytozome.net/search.php?show=text&method=Org_Creinhardtii>


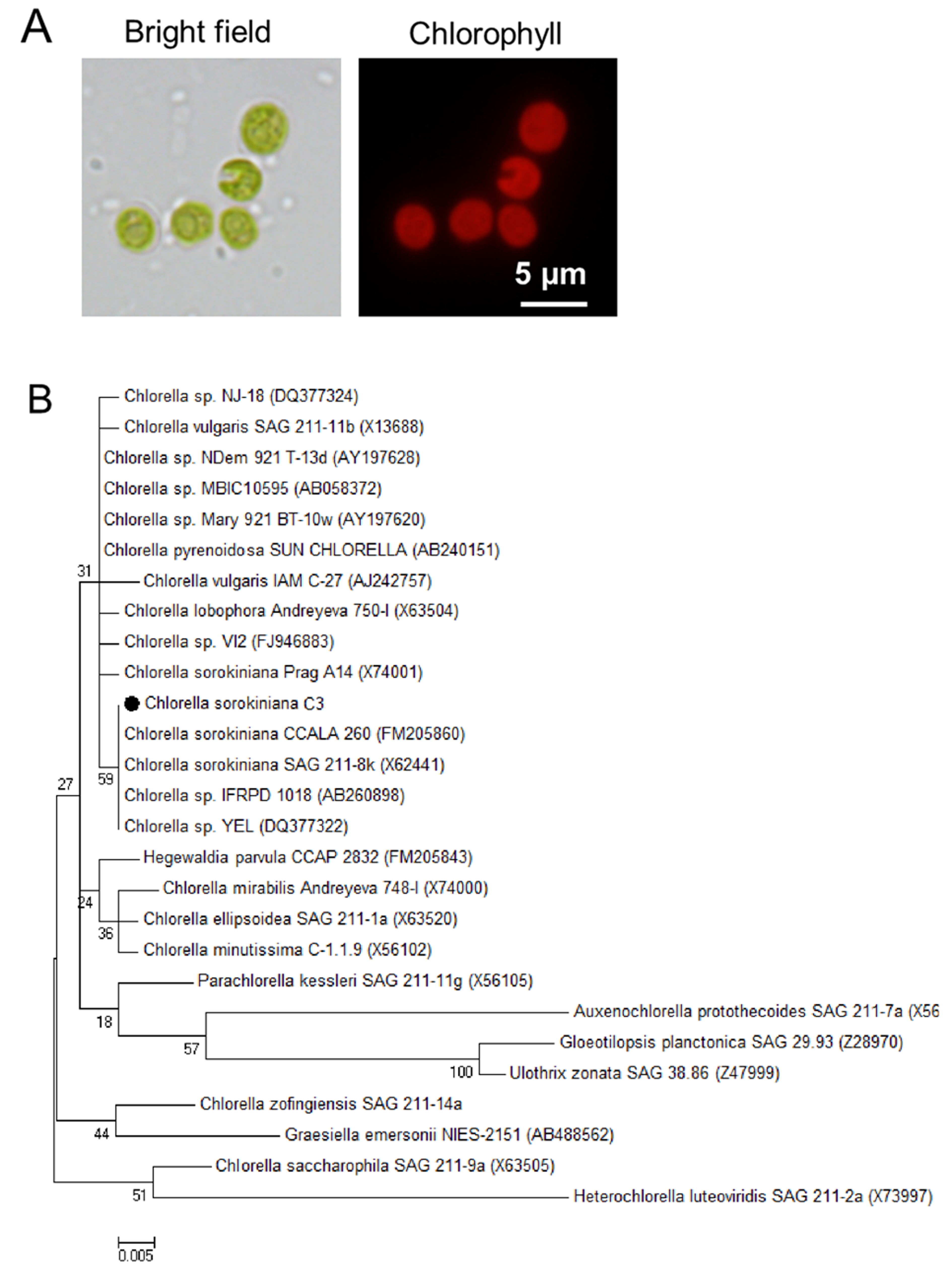


**Figure S1.** The identification of *Chlorella sorokiniana* C3.
